# The Photoprotective Effect of *S*-Methylmethionine Sulfonium in Skin

**DOI:** 10.3390/ijms160817088

**Published:** 2015-07-28

**Authors:** Won-Serk Kim, Hyun-Min Seo, Wang-Kyun Kim, Joon-Seok Choi, Ikyon Kim, Jong-Hyuk Sung

**Affiliations:** 1 Department of Dermatology, Kangbuk Samsung Hospital, Sungkyunkwan University School of Medicine, Seoul 110-746, Korea; E-Mails: susini@naver.com (W.-S.K.); blackshm@nate.com (H.-M.S.); 2 College of Pharmacy, Yonsei University, Incheon 406-840, Korea; E-Mails: noedark@nate.com (W.-K.K.); ikyonkim@yonsei.ac.kr (I.K.); 3 College of Pharmacy, Catholic University of Daegu, Gyeongbuk 712-702, Korea; E-Mail: joonschoi@naver.com

**Keywords:** *S*-methylmethionine sulfonium, UVB protection, dermal fibroblasts, keratinocyte progenitor cells, erythema

## Abstract

*S*-Methylmethionine sulfonium (SMMS) was reported to have wound-healing effects; we therefore have investigated the photoprotective effect of SMMS in the present study. SMMS increased the viability of keratinocyte progenitor cells (KPCs) and human dermal fibroblasts (hDFs) following ultraviolet B (UVB) irradiation, and reduced the UVB-induced apoptosis in these cells. SMMS increased the phosphorylation of extracellular signal-regulated kinases (ERK), and the inhibitor of the mitogen-activated protein kinase pathway significantly decreased the SMMS-induced viability of KPCs and hDFs. In addition, SMMS attenuated the UVB-induced reactive oxygen species (ROS) generation in KPCs and hDFs. SMMS induced the collagen synthesis and reduced the matrix metalloproteinase-1 expression in UVB-irradiated hDFs. In animal studies, application of 5% and 10% SMMS before and after UVB-irradiation significantly decreased the UVB-induced erythema index and depletion of Langerhans cells. In summary, SMMS protects KPCs and hDFs from UVB irradiation, and reduces UVB-induced skin erythema and immune suppression. Therefore, SMMS can be used as a cosmetic raw material, and protect skin from UVB.

## 1. Introduction

Ultraviolet (UV) light is a carcinogenic component of sunlight and widely known as one of the most relevant risk factors for skin cancers [1,2]. Irradiation of the skin with ultraviolet B (UVB) (wavelength, 290–320 nm) causes the photochemical reactions including the direct induction of DNA damage and the subsequent gene mutation that may lead to the development of skin cancer, or indirect secondary damage to skin via reactive oxygen species (ROS) generation [3,4,5]. In addition, UVB irradiation induces skin erythema, and changes the morphology and function of epidermal Langerhans cells (LCs). For example, Taguchi *et al.* reported that UVB irradiation induced the migration of epidermal LCs to the draining lymph nodes, resulting in a depletion of the epidermal LCs [6].

*S*-Methylmethionine sulfonium (SMMS), generally known as vitamin U, is effective in the treatment of injuries or ulcerations in the digestive tract and skin [7,8,9]. In addition, the anti-inflammatory, anti-depressant and cytoprotective effects of SMMS have been reported [10,11,12]. In skin, Kim *et al.* first reported that SMMS promoted the growth of human dermal fibroblasts (hDFs) as well as the migration of hDFs, therefore, accelerated wound healing in an animal model [8]. In addition, the activation of extracellular receptor kinase 1/2 (ERK1/2) mediated the SMMS-induced proliferation and migration of hDFs [8]. Therefore, SMMS has been used as a cosmetic raw material, but UVB-protective effect has not yet been investigated.

Herein, we first investigated the *in vitro* and *in vivo* photoprotective potential of SMMS in skin. To test the efficacy of SMMS, we evaluated the cell viability and proliferation of the keratinocyte progenitor cells (KPCs) and hDFs after exposure to UVB. In addition, signaling pathways and underlying molecular mechanisms are investigated in KPCs and hDFs. The *in vivo* photoprotective effect of SMMS was evaluated in hairless rats by quantitatively measuring the UVB-induced skin erythema and by conducting an immunohistochemical analysis of the depletion of epidermal LCs after UVB irradiation.

## 2. Results and Discussion

### 2.1. Photoprotective Effect of S-Methylmethionine Sulfonium (SMMS) in Keratinocyte Progenitor Cells (KPCs)

**Figure 1:**
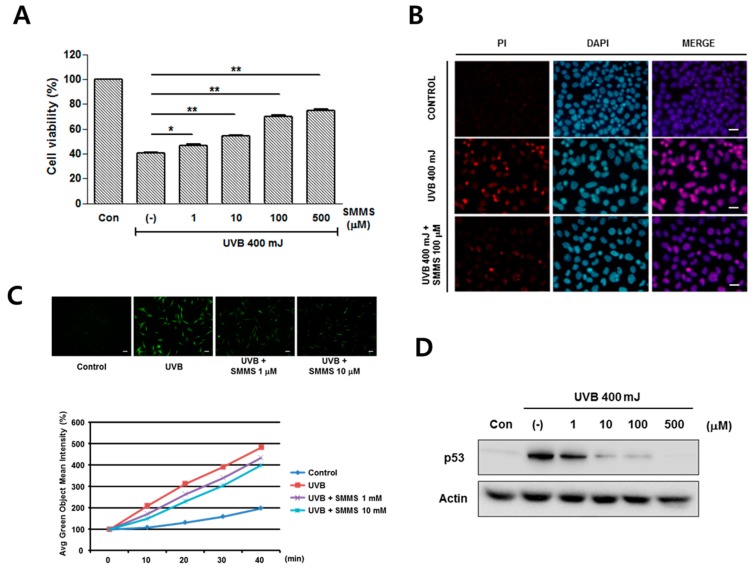

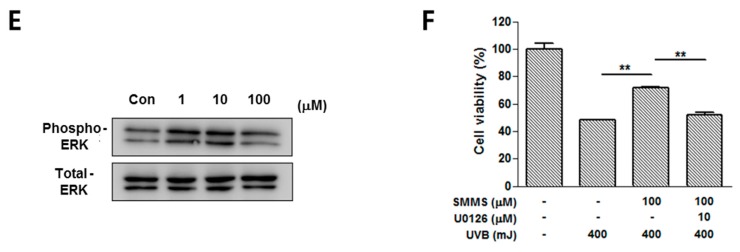
Photoprotective effect of *S*-methylmethionine sulfonium (SMMS) in keratinocyte progenitor cells (KPCs). (**A**) Cell viability of KPCs was measured in the presence of 400 mJ ultraviolet B (UVB) using the MTT (3-(4,5-dimethylthiazol-2-yl)-2,5-diphenyltetrazolium bromide) assay, and SMMS significantly increased the KPC viability from UVB irradiation. Con stands for unirradiated control, (-) stands for UVB-irradiated control, *n =* 3; (**B**) In a fluorescence staining of propidium iodide (PI), treatment of KPCs with 100 μM of SMMS reduced the PI-stained KPCs compared to UVB-irradiated control (*n =* 1). Scale bars = 100 μM; (**C**) SMMS treatment (1 and 10 μM) reduced the UVB-induced reactive oxygen species (ROS) generation in KPCs (*n =* 1). Scale bars = 100 μM; (**D**) Protein levels of p53 were detected using Western blotting, and SMMS reduced UVB-induced p53 level. (-) stands for UVB-irradiated control; (**E**) SMMS increased the levels of phosphorylated extracellular receptor kinase 1/2 (ERK1/2); (**F**) Pharmacological inhibition of ERK1/2 (U0126) significantly inhibited the SMMS-induced survival of KPCs (*n* = 3). * *p* < 0.05, ** *p* < 0.01.

The protective effects of SMMS in KPCs are showed in Figure 1. Firstly, we monitored the proliferation of KPCs treated with various concentrations of the SMMS (1, 10, 100, 500, and 1000 μM), but there is no difference in KPC proliferation (data not shown). On the other hand, in the presence of 400 mJ UVB, SMMS significantly increased the viability of KPCs in a dose-dependent manner compared to non-irradiated and UVB-irradiated controls (Figure 1A, *p* < 0.05).

In a fluorescence staining of propidium iodide (PI), treatment of KPCs with 100 μM of SMMS reduced the PI-stained KPCs compared to UVB-irradiated control. This result indicates that SMMS can protect KPCs from UVB-induced apoptosis (Figure 1B, *n* = 1). We investigated the protective mechanism of SMMS in KPCs from UVB irradiation, and found that SMMS (1 and 10 μM) reduced the UVB-induced reactive oxygen species (ROS) generation in KPCs (Figure 1C, *n* = 1).

Western blotting results in KPCs showed that the protein expression of p53 was induced by UVB irradiation, but it was dose-dependently reduced by the treatment of SMMS (Figure 1D). Regarding the signaling pathway, SMMS increased the levels of phosphorylated ERK in a dose-dependent manner (Figure 1E). Therefore, we carried out a pharmacological inhibition study using U0126 (selective inhibitor of mitogen-activated protein kinase (MAPK)/ERK kinase). U0126 significantly inhibited the SMMS-induced viability of KPCs, indicating that the MAPK pathway is involved in photoprotection of SMMS (Figure 1F, *p* < 0.01).

### 2.2. Photoprotective Effect of SMMS in Human Dermal Fibroblasts (hDFs)

**Figure 2:**
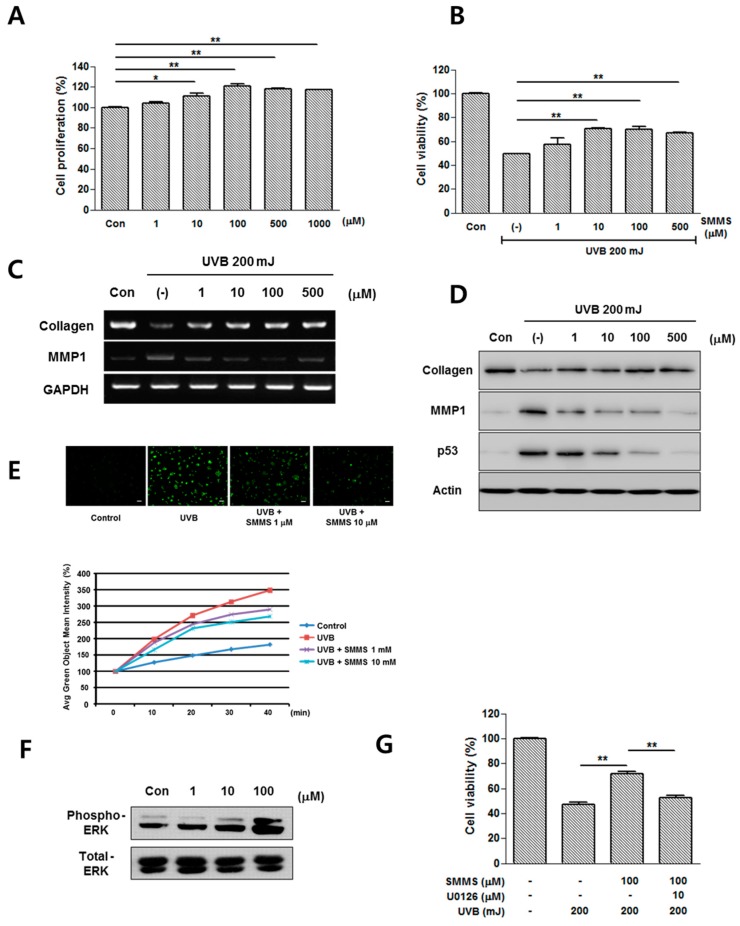
Photoprotective effects of SMMS in human dermal fibroblasts (hDFs). (**A**) SMMS increased the proliferation of hDFs. *n =* 3; (**B**) In addition, cell viability of hDFs was measured in the presence of 200 mJ UVB using MTT assay, and SMMS significantly increased the survival of hDFs. Con stands for unirradiated control, (-) stands for UVB-irradiated control, *n =* 3; (**C**) The mRNA expression of type I collagen and matrix metalloproteinase 1 (MMP-1) in hDFs was measured by RT-PCR 24 h after SMMS treatment; (**D**) The protein levels of type I collagen, MMP-1, and p53 were detected by using Western blotting; (**E**) SMMS treatment (1 and 10 μM) reduced the UVB-induced ROS generation in KPCs (*n =* 1). Scale bars = 100 μM; (**F**) SMMS significantly increased the levels of phosphorylated ERK1/2 in a dose-dependent manner; (**G**) Pharmacological inhibition of ERK1/2 (U0126) significantly inhibited the SMMS-induced survival of hDFs (*n =* 3). * *p* < 0.05, ** *p* < 0.01.

Figure 2 shows the photoprotective effect of SMMS in hDFs. Proliferation of hDFs was slightly increased by SMMS (10–1000 μM, Figure 2A, *p* < 0.05) as reported previously [8]. In addition, SMMS increased the viability of UVB-irradiated hDFs in a dose-dependent manner compared to UVB-irradiated control (200 mJ UVB, Figure 2B, *p* < 0.01).

The mRNA expression of type I collagen and MMP-1 in hDFs was measured by RT-PCR 24 h after SMMS treatment (Figure 2C). Although UVB irradiation reduced the mRNA expression of type I collagen, SMMS increased the mRNA expression of type I collagen in UVB-irradiated hDFs. In addition, SMMS decreased UVB-induced MMP-1 mRNA level (Figure 2C). The protein levels of type I collagen and MMP-1 were also measured using Western blotting, and showed a similar trend in a dose-dependent manner. In addition, UVB-induced protein levels of p53 were dose-dependently decreased by SMMS (Figure 2D). We investigated the protective mechanism of SMMS in hDFs from UVB irradiation, and found that SMMS (1 and 10 mM) reduced the UVB-induced ROS generation in hDFs (Figure 2E, *n =* 1).

SMMS also increased the levels of phosphorylated ERK in hDFs (Figure 2F). In pharmacological inhibition studies, U0126 significantly attenuated the SMMS-induced viability of hDFs, indicating that the MAPK pathway is involved in hDF protection (Figure 2G, *p* < 0.01). These results are similar to a previous report that SMMS mediates mitogenic effects through the MAPK pathway in hDFs [8].

### 2.3. Protective Effect of SMMS in Ultraviolet B-Induced Erythema

**Figure 3:**
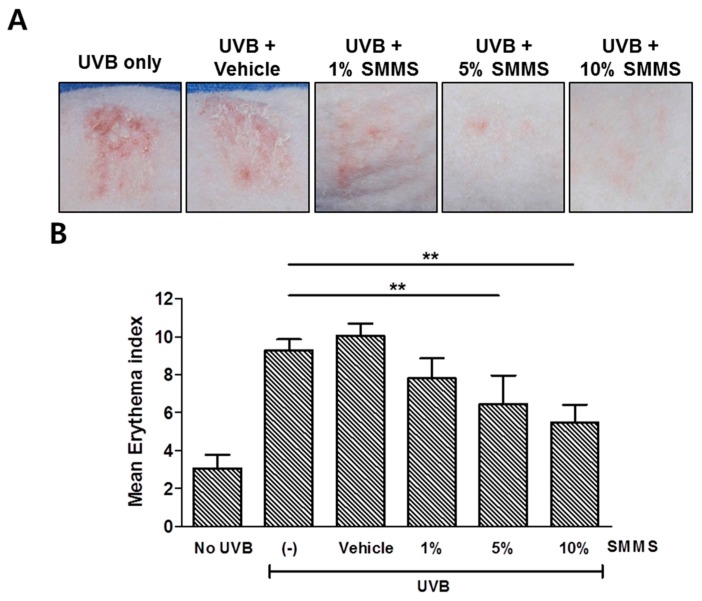
Photoprotective effect of SMMS in hairless albino rats. Erythema was induced by UVB irradiation (2× minimal erythema dose (MED)) on the backs of hairless albino rats. Before and immediately after UVB irradiation, we applied topically 1%, 5%, and 10% SMMS mixture to the right side of three rats, respectively (*n =* 3 for each group). (**A**) The photographs show the rats’ skin 72 h after UVB irradiation. Scale bars = 1 cm; (**B**) Erythema index was measured using spectrophotometry 72 h after ultraviolet B irradiation. Application of 5% and 10% SMMS significantly reduced the UVB-induced skin erythema. (-) stands for UVB-irradiated control. All values are presented as a mean ± standard deviation. ** *p* < 0.01.

The topical application of 5% (31.25 mM) and 10% (62.5 mM) SMMS significantly reduced the degree of erythema compared to the UVB-irradiated control group in photographs (Figure 3A). Therefore, spectrophotometric measurements were performed; topical application of 5% and 10% SMMS 30 min before and immediately after UVB-irradiation significantly decreased the erythema index in hairless albino rats (Figure 3B, *p* < 0.01).

### 2.4. Protective Effect of SMMS in UVB-Induced Depletion of Epidermal Langerhans Cells (LCs)

**Figure 4:**
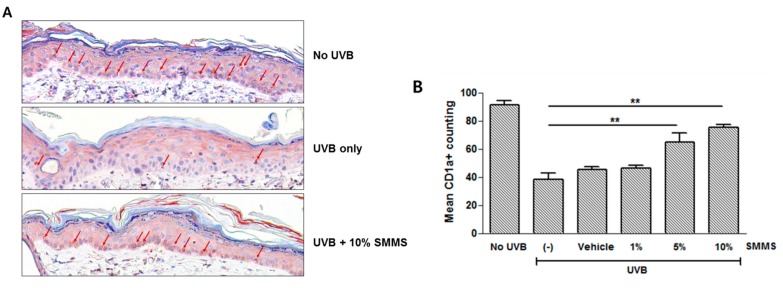
Immunohistochemical staining of cluster of differentiation 1a (CD1a) for epidermal Langerhans cells (LCs). (**A**) The depletion of epidermal LCs was measured via a CD1a staining 72 h after UVB irradiation (2× MED). Arrow indicates CD1a^+^ cells. Scale bars = 100 µm; (**B**) Application of 5% and 10% SMMS significantly reduced the UVB-induced LC depletion. (-) stands for UVB-irradiated control. All values are presented as a mean ± STD (*n =* 3). ** *p* < 0.01.

Figure 4 shows the immunohistochemical staining of cluster of differentiation 1a (CD1a) for epidermal LCs, and depletion of epidermal LCs was measured via a CD1a staining 72 h after UVB irradiation (Figure 4A). Calculation of immunohistochemical results showed the decreased CD1a^+^ LC cells in UVB-irradiated control (UVB only), vehicle-treated (UVB + Vehicle), and 1% SMMS treatment groups (UVB + 1% SMMS). However, topical application of 5% and 10% SMMS significantly attenuated the UVB-induced LC depletion. (Figure 4B, *p* < 0.01, *n =* 3).

### 2.5. Discussion

We investigated the UVB-protective effects and underlying mechanisms of SMMS in skin. Firstly, MTT assay showed that the UVB-reduced viability of KPCs and hDFs was significantly attenuated in the presence of SMMS. PI-stained KPCs were reduced by SMMS compared to irradiated controls, suggesting that SMMS can prevent UVB-induced apoptosis. SMMS decreased the protein level of p53 and increased that of phosphorylated ERK in both KPCs and hDFs. Furthermore, the protein level and mRNA expression of type I collagen was increased, and that of MMP-1 was decreased by SMMS in hDFs. When we pharmacologically inhibited the ERK1/2 pathway, the viability of KPCs and hDFs was significantly decreased. *In vivo* study in hairless albino rats showed that applying 5% and 10% SMMS before and immediately after UVB-irradiation significantly decreased skin erythema and prevented UVB-induced depletion of LCs. Collectively, our results suggest that SMMS has UVB-protective effects in skin.

We first demonstrated the signaling pathways and molecular mechanisms underlying photoprotective effects of SMMS in the present study. We found that ERK1/2 activation by SMMS is responsible for increasing the viability of KPCs and hDFs, since a blockade of the ERK1/2 pathway significantly attenuated the viability of both KPCs and hDFs. In a previous study, SMMS increased the proliferation of hDFs in a skin wound model, and the cellular proliferation was associated with the up-regulation of MEK1/2 following ERK1/2 signaling [8]. On the other hand, the UVB can induce apoptosis in skin, which is mediated through the action of p53 [2,13,14,15]. In our study, the protein levels of p53 after UVB were dose-dependently decreased by the treatment of SMMS in both KPCs and hDFs. In addition to MAPK pathway activation, down-regulation of p53 may contribute to inhibit the apoptosis of skin cells from UVB irradiation.

The most visually apparent indicator of UVB-induced skin inflammation is erythema. Increased blood flow in the superficial and deep vascular plexus of the skin is responsible for the erythema. It usually appeared several hours after UVB irradiation. It reaches a maximum intensity after 12 to 24 h. However, when the dose of irradiation is high, erythema is more persistent and intense, and the response is likely to include pain, edema or swelling [16,17]. In our study, we demonstrated that a topical application containing 5% and 10% SMMS significantly decreased the EI measured by spectrophotometry when compared to the irradiated controls (*p* < 0.05). This result suggests that applying SMMS can prevent UVB-induced skin erythema.

UV exposure alters the epidermal concentration, morphology and function of LCs, which can elicit cutaneous immunosuppressive responses [6,18]. After UV-irradiation, LCs begin to migrate from the epidermis to draining lymph nodes without functional maturity. Immature LCs activate Th2 cells, which generates suppressor T cells [19]. In our study, we demonstrated that a topical application containing 5% and 10% SMMS significantly decreased the depletion of epidermal CD1a^+^ LCs when compared to the UVB-irradiated controls. This suggests that the application of SMMS can prevent the UVB-induced suppression of the cutaneous immune system.

## 3. Experimental Section

### 3.1. Cell Culture

KPCs were obtained from CELLnTEC (Bern, Switzerland), and were grown in Keratinocyte Growth Medium 2 with supplement mix (KGM2, PromoCell, Heidelberg, Germany) at 37 °C in 5% CO_2_. KPCs were characterized by flow cytometry analysis using antibodies to integrin α6 and cluster of differentiation 71 (CD 71) as described in a previous study [20]. hDFs were isolated as described previously [21], and grown in Dulbecco’s Modified Eagle’s Medium (DMEM) of low glucose (Hyclone, Thermo Scientific, Logan, UT, USA). We added 10% fetal bovine serum (Gibco, Invitrogen, Carlsbad, CA, USA) and 1% Penicillin and Streptomycin (Gibco) at 37 °C in a humidified atmosphere containing 5% CO_2_ for subculture. We replaced the media of hDFs and KPCs every two days and we performed a subculture every three days.

### 3.2. Proliferation and Viability Assay

KPCs (4 × 10^4^/well) and hDFs (3 × 10^4^/well) were seeded in 6-well plates in the KGM2 and DMEM with 10% FBS. The media were changed to the Keratinocyte Basal Medium 2 (KBM2) or DMEM without serum overnight. After starvation, the cells were treated with various concentrations of SMMS in the presence or absence of UVB-irradiation. Then, cells were incubated in KBM2 or DMEM for 48 h. Then, we performed the MTT assay. The MTT solution (5 mg/mL in PBS) was added to each well at 5% of the media’s volume. The cells were incubated at 37 °C for 2 h, and the supernatant was removed. Dimethyl sulfoxide was then added in order to dissolve the formazan crystals, and the absorbance was measured at 595 nm using an ELISA reader (TECAN, Grodig, Austria).

### 3.3. Immunocytochemistry

The adherent cells on the slide were fixed with 1% paraformaldehyde (*v*/*v*) in 1× PBS and placed on ice for 30 min. After washing the cells twice with 1× PBS, we added ice-cold 70% (*v*/*v*) ethanol into the cells and kept the cells for at least 30 min. The cells were washed with 1× PBS twice and treated with a PI/RNAse buffer (1:10) (Invitrogen) under room temperature condition for 30 min. In addition, we used 4′,6-diamidino-2-phenylindole (DAPI, 1:1000) to counterstain the cells at room temperature for 10 min.

### 3.4. Cellular Reactive Oxygen Species (ROS) Generation Assay

ROS generation was measured using 2′,7′-dichlorodihydrofluorescein diacetate (DCF-DA, Molecular Probes, Eugene, OR, USA). Cells were seeded on six well plate in 0.2% fetal bovine serum (FBS) in α-minimum essential medium (MEM) medium. UVB-irradiated cells were co-treated with SMMS (10 μM) and 20 μM DCF-DA. Each well was photographed every 10 min under standard incubation conditions using an IncuCyte™ (Essen Bioscience, Ann Arbor, MI, USA) inside an incubator. Fluorescence intensity of DCF-DA was measured using the IncuCyte™ software.

### 3.5. Western Blot Analysis

Cells (2 × 10^5^ cells/mL) were seeded into a 60 mm-dish for 1 day to achieve 80% confluence, and treated according to each reaction condition. The cells were lysed with a 1× radioimmunoprecipitation assay buffer (50 mM Tris–HCl, 0.15 M NaCl, 1 mM ethylenediaminetetraacetic acid (EDTA), 1% Triton-X100, pH 7.4, 1% sodium dodecyl sulfate (SDS), 50 mM NaF, 1 mM Na_3_VO_4_, 5 mM Dithiothreitol, 1 mg/mL Leupeptin and 1 mM phenylmethylsulfonyl fluoride). Each 40 μg of sample protein was separated into 10%–12% SDS–polyacrylamide gel by electrophoresis. Proteins were transferred to polyvinylidene fluoride membranes and incubated with antibodies of phospho-ERK1/2 (1:2000; mouse source), total-ERK1/2 (1:4000; rabbit source), phospho-p53 (1:1000; rabbit source), Collagen (1:1000 rabbit source), and matrix metalloproteinase-1 (MMP-1) (1:1000 rabbit source). Then, membranes were washed and incubated with horseradish peroxidase conjugated anti-mouse IgG (Santa Cruz Biotechnology, Santa Cruz, CA, USA) and anti-rabbit IgG antibody (Santa Cruz). Blots were reacted with immobilon western reagent (ECL; Millipore, Billerica, MA, USA) and exposed to an X-ray film.

### 3.6. Reverse Transcription-Polymerase Chain Reaction (RT-PCR)

The total RNA of hDFs was extracted with Trizol reagent followed by a reverse transcription to cDNA. The following oligonucleotides were used as primers: collagen type I (5′-TAGGGTCTAGACATGTTCAGCTTTGT-3′ and 5′-GTGATTGGTGGGATGTCTTCGT-3′), MMP-1 (5′-AGATGTGGAGTGCCTGATGT-3′ and 5′-AGCTAGGGTACATCAAAGCC-3′), and the control glyceraldehyde 3-phosphate dehydrogenase (GAPDH) (5′-CGAGATCCCTCCAAAATCAA-3′ and 5′-TGTGGTCATGAGTCCTCCCA-3′). The polymerase chain reaction (PCR) was carried out in a total volume of 30 μL for PCR amplification of cDNA, which was reverse-transcribed from the total RNA. After initial denaturation at 95 °C for 5 min, amplification was performed in 35 cycles; for example, 30 s at 95 °C, 20 s at 54 °C, and 30 s at 72 °C. These cycles were followed by a final extension at 72 °C for another 10 min. The GAPDH mRNA level was used for sample standardization.

### 3.7. Animal Study

*In vivo* experiments were performed on 8-week-old, female hairless albino rats (HWY/Slc, Japan SLC Inc., Shizuoka, Japan) ranging in weight from 220 to 260 g. The animals were housed in a temperature-controlled room with free access to water and standard laboratory food. They were housed under special pathogen-free conditions in cages with a 12 h light and 12 h dark cycle. Each animal was used only once. All animal experiments were reviewed by the Animal Care and Use Committee in Kangbuk Samsung Hospital according to the NIH guidelines of the Principles of Laboratory Animal Care (IACUC-201407069, 31 July 2014). The animals were divided into six groups (*n =* 3 for each group): (1) non-irradiated control; (2) UVB-irradiated control; (3) treated with the vehicle (100% pure petrolatum) and UVB-irradiated; and (4–6) each treated with various concentrations of SMMS (1%, 5% and 10% *w*/*w*) and then UVB-irradiated. The three concentrations of SMMS were formulated in a vehicle containing pure petrolatum.

### 3.8. UV Source and Irradiation Protocol

The irradiation source used in this study was a targeted broadband UVB device (DuaLight™, TheraLight Inc., Carlsbad, CA, USA) with a high-pressure mercury lamp. UV radiation of this light source is delivered through a 1.9 × 1.9 cm-sized square aperture. The UVB spectral output includes peaks at 302 and 312 nm, with an average weighted erythemal wavelength (*i.e.*, the mean value of total area about spectrum and intensity of the device) of 304 nm. The hairless albino rats underwent standard minimum erythema dose (MED) testing. Twenty-four hours later, MED was determined at the erythematous skin exposed to the shortest duration of UVB. Then, six areas on the backs of the rats were chosen for each group. Each test agent was applied and allowed to dry for 30 min prior to UVB irradiation with 2× MED. A repeat application was performed immediately after UVB irradiation.

### 3.9. Spectrophotometric Measurements

Seventy-two hours later, UVB-irradiated sites or non-irradiated controls were analyzed via spectrophotometry (DSM II ColorMeter^®^, Cortex technology, Hadsund, Denmark) to measure the degree of erythema quantitatively. The spectrophotometry sends white light into the skin and measures the color of the reflection of light from the skin. The color is measured by a color detection chip inside the probe. The color-detection chip measures the following narrow bands of light wavelengths using color filters:

Ig: reflected intensity at 620 ± 30 nm; Ir: reflected intensity at 550 ± 30 nm.
Then, the erythema index (EI) can be calculated as describe previously [22]: 100 × log(Ir/Ig).

### 3.10. Immunohistochemical Analysis

To detect CD1a^+^ stained LCs, skin specimens were taken 72 h after UVB irradiation. A total of 36 specimens (six 3 mm-punch biopsies for each group) were obtained. Tissue samples were fixed with a 10% formalin neutral buffered solution, embedded in polyester wax for immunostaining using anti-CD1a (1:200) for epidermal LCs, and analyzed using a direct microscopic count of positive cells per 200× field in 3 mm specimens.

### 3.11. Statistical Analysis

Statistical significance was determined using Mann-Whitney U (Wilcoxon rank sum) test. The results were expressed as mean ± standard deviations. *p* < 0.05 and *p* < 0.01 was considered as statistically significant. Statistical analysis was performed using SPSS 18.0 (SPSS, IBM Corp, Armonk, NY, USA).

## 4. Conclusions

This study provides evidence of the photoprotective effects of SMMS, which protects KPCs and hDFs from UVB irradiation through activating the MAPK pathway. Moreover, this is the first *in vivo* demonstration that SMMS reduces UVB-induced skin erythema and immune suppression. Therefore, SMMS can be considered for use as a cosmetic raw material able to protect skin from UVB irradiation.
